# Parliament and the Profession

**Published:** 1919-05-17

**Authors:** 


### Parliament and the Profession.

### Queen Mary's Hospital, Stratford.

SIR L. WORTHINGTON EVANS stated that in view of the difficulty in obtaining hospital accommodation for discharged soldiers in the West Ham district, arrangements have been completed for the treatment of men at Queen Mary's Hospital, Stratford, and it is anticipated that this provision will be adequate.

### Inadequacy of Nursing Service.

ANSWERING a question by Sir K. Wood as to the alleged inadequacy of the nursing service throughout the country, Major Astor stated that grants from the Exchequer to Insurance Committees in aid of the provision of nursing services for insured persons were voted by Parliament just before the outbreak of war. The inauguration of these services has been deferred in consequence of war conditions. The question of renewing those arrangements, or of including them in a wider provision for the whole community, is of great importance for the health of the people, and is amongst the matters that are being carefully considered in connection both with possible developments of insurance services and health services generally.

### Leprosy in India.

SIR J. D. REES asked the Secretary of State for India whether the lepers sent to a leper asylum by magisterial order are only detained so long as they suffer from an open sore; whether such detention is a vain expenditure of public money, in view of the fact, believed to be established, that leprosy is contagious in all its stages; and whether any amendment of the Leper Act is contemplated in order to check the spread of this disease in India. Mr. Montagu replied that the answer to the first part of the question is in the affirmative. He was advised that medical opinion is divided on the question whether leprosy is contagious in all its stages. He was not aware that an amendment of the Indian Leper Act is in contemplation.

